# Evaluation of the efficacy of a trivalent vaccine mixture against a triple challenge with *Mycoplasma hyopneumoniae*, PCV2, and PRRSV and the efficacy comparison of the respective monovalent vaccines against a single challenge

**DOI:** 10.1186/s12917-019-2091-6

**Published:** 2019-10-16

**Authors:** Taehwan Oh, Kee Hwan Park, Siyeon Yang, Jiwoon Jeong, Ikjae Kang, Changhoon Park, Chanhee Chae

**Affiliations:** College of Veterinary Medicine, Department of Veterinary Pathology, Seoul National University, Gwanak-ro 1, Gwanak-gu, Seoul, 08826 Republic of Korea

**Keywords:** *Mycoplasma hyopneumoniae*, Porcine circovirus type 2, Porcine reproductive and respiratory syndrome virus, Porcine respiratory disease complex

## Abstract

**Background:**

The objective of this study was to assess the efficacy of a trivalent vaccine mixture and compare it to the respective monovalent vaccines against *Mycoplasma hyopneumoniae*, porcine circovirus type 2 (PCV2), and porcine reproductive and respiratory syndrome virus (PRRSV).

**Results:**

Pigs that were triple challenged with *M. hyopneumoniae*, PCV2, and PRRSV following vaccination with the trivalent vaccine mixture exhibited a significantly better growth performance when compared to unvaccinated and challenged pigs. A statistical difference was not found when comparing pig populations which were vaccinated with the trivalent vaccine followed by a triple challenge and pigs vaccinated with monovalent *M hyopneumoniae* vaccine followed by mycoplasmal single challenge in the following areas: *M. hyopneumoniae* nasal shedding, the number of *M. hyopneumoniae*-specific interferon-γ secreting cells (IFN-γ-SC), and mycoplasmal lung lesion scores. Pigs vaccinated with the trivalent vaccine mixture followed by a triple challenge resulted in a similar reduction of PCV2 viremia, an increase in the number of PCV2-specific IFN-γ-SC and reduction in interstitial lung lesion scores when compared to pigs vaccinated with a PCV-2 vaccine and challenged with PCV2 only. Lastly, there was a significant difference in the reduction of PRRSV viremia, an increase in PRRSV-specific IFN-γ-SC and a reduction of interstitial lung lesion scores between pigs vaccinated with the trivalent vaccine mixture followed by a triple challenge and pigs vaccinated with a monovalent PRRSV vaccine followed by PRRSV challenge only.

**Conclusion:**

The trivalent vaccine mixture was efficacious against a triple challenge of *M. hyopneumoniae*, PCV2, and PRRSV. The trivalent vaccine mixture, however, did not result in equal protection when compared against each respective monovalent vaccine, with the largest vaccine occurring within PRRSV.

## Background

Porcine respiratory disease complex (PRDC) is a disease that predominately affects growing to finishing pigs between the ages of 14 to 20 weeks. This is commonly referred to as the ‘18-week wall’ in modern commercial pig production. There are multiple factors that contribute to PRDC including multiple viral and bacterial infections, environmental conditions, and management practices. Clinical signs are characterized by slow and uneven growth, decreased feeding efficiency, anorexia, fever, cough, and dyspnea [1, 2].

Currently, PRDC is one of the biggest health concerns to the Asian swine industry. The etiological agents associated with PRDC may vary between different geographic regions, but in Asia, *Mycoplasma hyopneumoniae*, porcine circovirus type 2 (PCV2), and porcine reproductive and respiratory syndrome virus (PRRSV) are the primary pathogens which cause the majority of the PRDC cases resulting in devastating economic losses [1]. Most Asian swine producers control PRDC by use of preventative vaccines rather than antibiotics. Combination vaccines are preferred in order to reduce pig stress and to decrease labor cost. A trivalent vaccine mixture (3FLEX, Boehringer Ingelheim Vetmedica, St. Joseph, Missouri, USA) against *M. hyopneumoniae*, PCV2, and PRRSV has been licensed in many Asian countries to control PRDC. However, there is concern from some swine producers that this trivalent vaccine mixture may be less effective compared to the respective monovalent vaccines currently available because of possible interferences among the mixed antigens. In this study, we decided to evaluate the efficacy of this trivalent vaccine mixture (3FLEX, Boehringer Ingelheim Vetmedica) against a triple challenge of *M. hyopneumoniae*, PCV2, and PRRSV and compare it to the efficacy of the respective monovalent vaccines. Clinical, immunological, microbiological, and pathological parameters were chosen for evaluation.

## Results

### Clinical observation

**Fig. 1:**
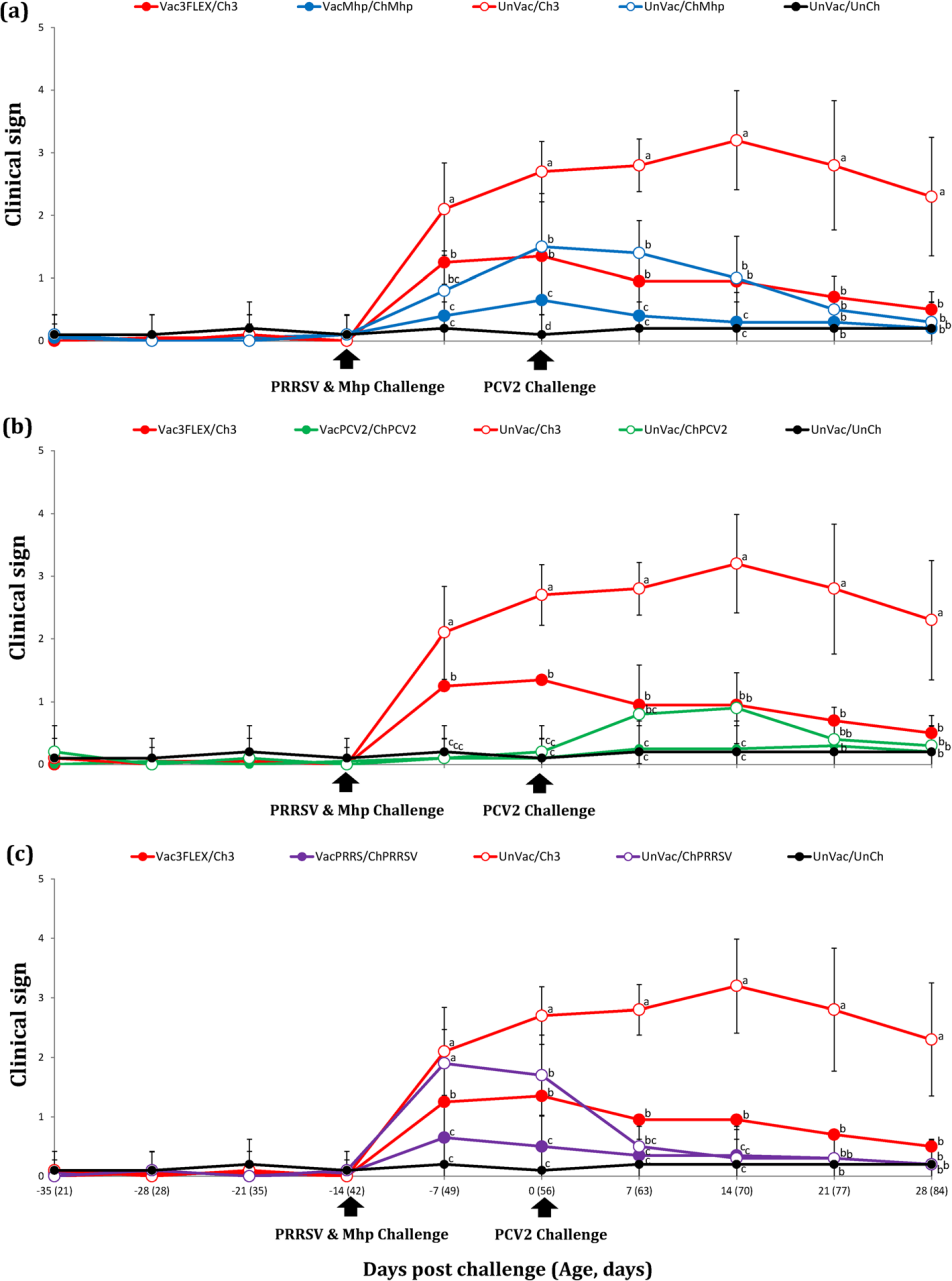
**a** Mean respiratory scores between trivalent vaccine mixture and respective monovalent *M. hyopneumoniae* vaccine. **b** Mean respiratory scores between trivalent vaccine mixture and respective monovalent PCV2 vaccine. **c** Mean respiratory scores between trivalent vaccine mixture and respective monovalent PRRS vaccine. Variation is expressed as the standard deviation. Different letters within a sampling point mean statistically significant differences (*P* < 0.05)

The mean scores for respiratory disease were significantly higher (*P* < 0.05) in pigs from the UnVac/Ch3 group when compared to the Vac3FLEX/Ch3, VacMhp/ChMhp, UnVac/ChMhp, and UnVac/UnCh groups from − 7 to 28 dpc (Fig. 1a) and Vac3FLEX/Ch3, VacPCV2/ChPCV2, UnVac/ChPCV2, and UnVac/UnCh groups from − 7 to 28 dpc (Fig. 1b). The mean respiratory scores were significantly higher (*P* < 0.05) in pigs from the UnVac/Ch3 and UnVac/ChPRRSV groups compared to the Vac3FLEX/Ch3, VacPRRS/ChPRRSV, and UnVac/UnCh groups at − 7 dpc (Fig. 1c).

### Average daily weight gain

No statistical difference was observed in the average body weight (mean ± standard deviation) among the 9 groups at 21 days of age which was the start of the study; Vac3FLEX/Ch3 (*n* = 20, 6.42 Kg ± 0.54), VacMhp/ChMhp (*n* = 20, 6.41 Kg ± 0.52), VacPCV2/ChPCV2 (*n* = 20, 6.41 Kg ± 0.49), VacPRRS/ChPRRSV (*n* = 20, 6.42 Kg ± 0.45), UnVac/Ch3 (*n* = 10, 6.41 Kg ± 0.51), UnVac/ChMhp (*n* = 10, 6.43 Kg ± 0.47), UnVac/ChPCV2 (*n* = 10, 6.41 Kg ± 0.45), UnVac/ChPRRSV (*n* = 10, 6.42 Kg ± 0.45) and UnVac/UnCh (*n* = 10, 6.42 Kg ± 0.49). The overall ADWG (from 21 to 84 days old) of pigs from the Vac3FLEX/Ch3, VacMhp/ChMhp, VacPCV2/ChPCV2, VacPRRS/ChPRRSV, UnVac/ChMhp, UnVac/ChPCV2, UnVac/ChPRRSV, and UnVac/UnCh groups was significantly higher (*P* < 0.05) when compared to the UnVac/Ch3 group (Table 1).

**Table 1:** Average daily weight gain (ADWG) in trivalent vaccine mixture and respective monovalent vaccines

Groups	Average Daily Weight Gain (grams/day/pig)		
	21–56	56–84	21–84
Vac3FLEX/Ch3	367.29 ± 23.18^ab^	654.29 ± 57.30^a^	494.84 ± 30.77^a^
VacMhp/ChMhp	375.71 ± 25.50^a^	663.93 ± 60.33^a^	503.81 ± 27.95^a^
UnVac/Ch3	343.43 ± 13.46^b^	537.86 ± 36.51^b^	429.84 ± 18.90^b^
UnVac/ChMhp	370.57 ± 25.31^ab^	643.57 ± 63.39^a^	491.91 ± 28.79^a^
UnVac/UnCh	386.57 ± 25.56^a^	667.50 ± 68.95^a^	511.43 ± 32.98^a^
Vac3FLEX/Ch3	367.29 ± 23.18^ab^	654.29 ± 57.30^a^	494.84 ± 30.77^a^
VacPCV2/ChPCV2	377.43 ± 28.30^a^	664.29 ± 71.48^a^	504.92 ± 27.92^a^
UnVac/Ch3	343.43 ± 13.46^b^	537.86 ± 36.51^b^	429.84 ± 18.90^b^
UnVac/ChPCV2	376.57 ± 24.12^a^	649.29 ± 64.66^a^	497.78 ± 32.30^a^
UnVac/UnCh	386.57 ± 25.56^a^	667.50 ± 68.95^a^	511.43 ± 32.98^a^
Vac3FLEX/Ch3	367.29 ± 23.18^abc^	654.29 ± 57.30^a^	494.84 ± 30.77^a^
VacPRRS/ChPRRSV	375.57 ± 26.78^ab^	660.71 ± 62.28^a^	502.30 ± 29.15^a^
UnVac/Ch3	343.43 ± 13.46^c^	537.86 ± 36.51^b^	429.84 ± 18.90^b^
UnVac/ChPRRSV	354.57 ± 25.00^bc^	645.36 ± 59.38^a^	483.81 ± 30.49^a^
UnVac/UnCh	386.57 ± 25.56^a^	667.50 ± 68.95^a^	511.43 ± 32.98^a^

Different letters mean statistically significant differences within 5 groups (*P* < 0.05)

### Quantification of 3 pathogens

**Fig. 2:**
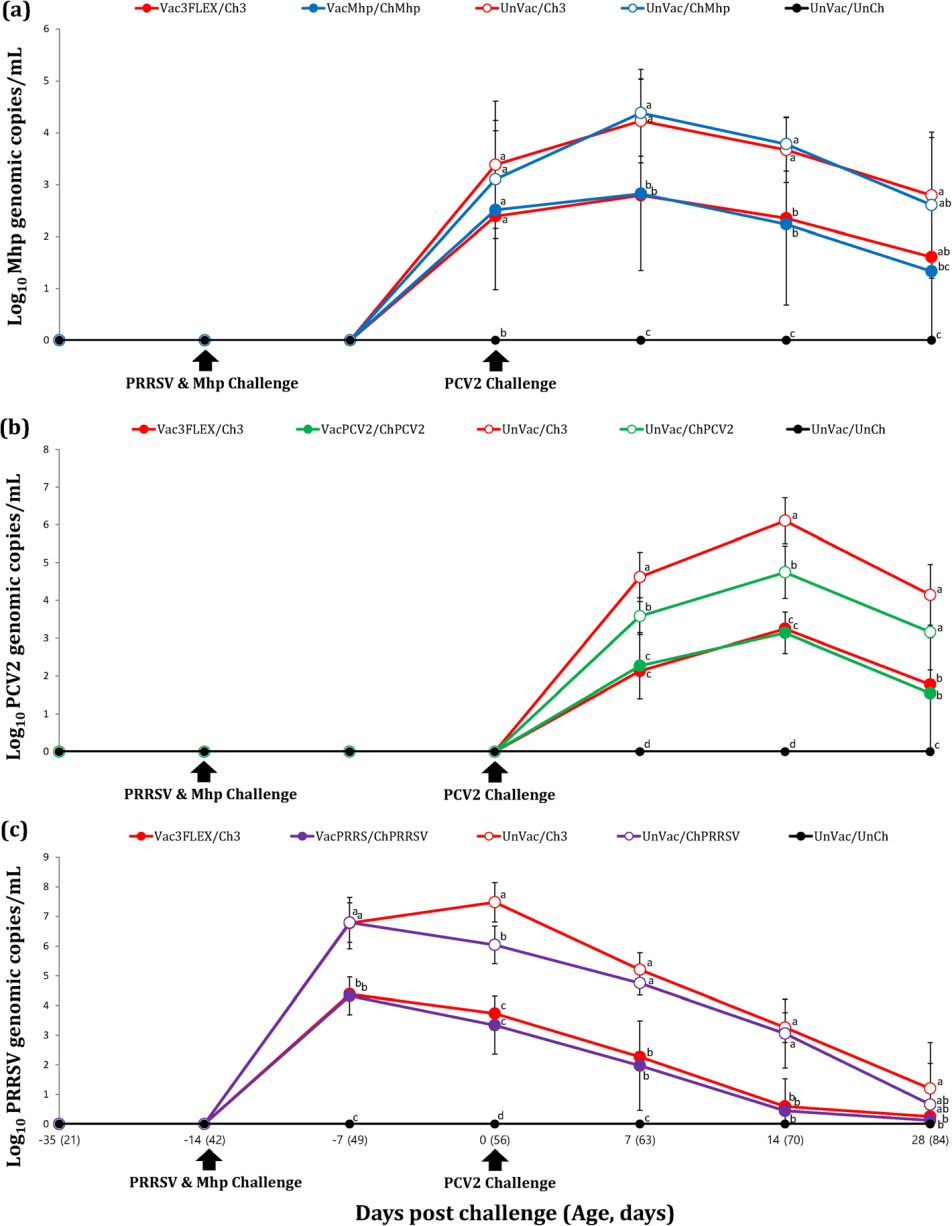
**a** Mean values of the genomic copy number of *M. hyopneumoniae* DNA in nasal swabs. **b** Mean values of the genomic copy number of PCV2 DNA in serum. **c** Mean values of the genomic copy number of PRRSV RNA in serum. Variation is expressed as the standard deviation. Different letters within a sampling point mean statistically significant differences (*P* < 0.05)

Prior to challenge, no genomic copies of *M. hyopneumoniae*, PCV2, and PRRSV were detected in any of the pigs from all 9 groups. Pigs in the Vac3FLEX/Ch3 and VacMhp/ChMhp groups had significantly less (*P* < 0.05) *M. hyopneumoniae* genomic copies in their nasal swabs compared to the UnVac/Ch3 and UnVac/ChMhp groups at 7 and 14 dpc. Pigs in the VacMhp/ChMhp group had a significantly lower (*P* < 0.05) number of *M. hyopneumoniae* genomic copies in their nasal swabs compared to the UnVac/Ch3 group at 28 dpc (Fig. 2a).

Pigs from the Vac3FLEX/Ch3 and VacPCV2/ChPCV2 groups had a significantly lower (*P* < 0.05) number of genomic copies of PCV2 in their blood compared to the UnVac/Ch3 and UnVac/ChPCV2 groups at 7, 14, and 28 dpc. Pigs from the UnVac/ChPCV2 group had a significantly lower (*P* < 0.05) number of genomic copies of PCV2 in their blood compared to the UnVac/Ch3 group at 7 and 14 dpc (Fig. 2b).

The Vac3FLEX/Ch3 and VacPRRS/ChPRRSV groups had a significantly lower (*P* < 0.05) number of genomic copies of PRRSV in their blood compared to the UnVac/Ch3 and UnVac/ChPRRSV groups at − 7, 0, 7, and 14 dpc. The VacPRRS/ChPRRSV group had a significantly lower (*P* < 0.05) number of genomic copies of PRRSV in their blood compared to the UnVac/Ch3 group at 28 dpc. The UnVac/ChPRRSV group had a significantly lower (*P* < 0.05) number of genomic copies of PRRSV in their blood compared to the UnVac/Ch3 group at 0 dpc. No genomic copies of PRRSV were detected in any of the pigs from UnVac/UnCh group (Fig. 2c). No genomic copies of *M. hyopneumoniae*, PCV2, and PRRSV were detected in any of the pigs from the UnVac/UnCh group.

### Serology

**Fig. 3:**
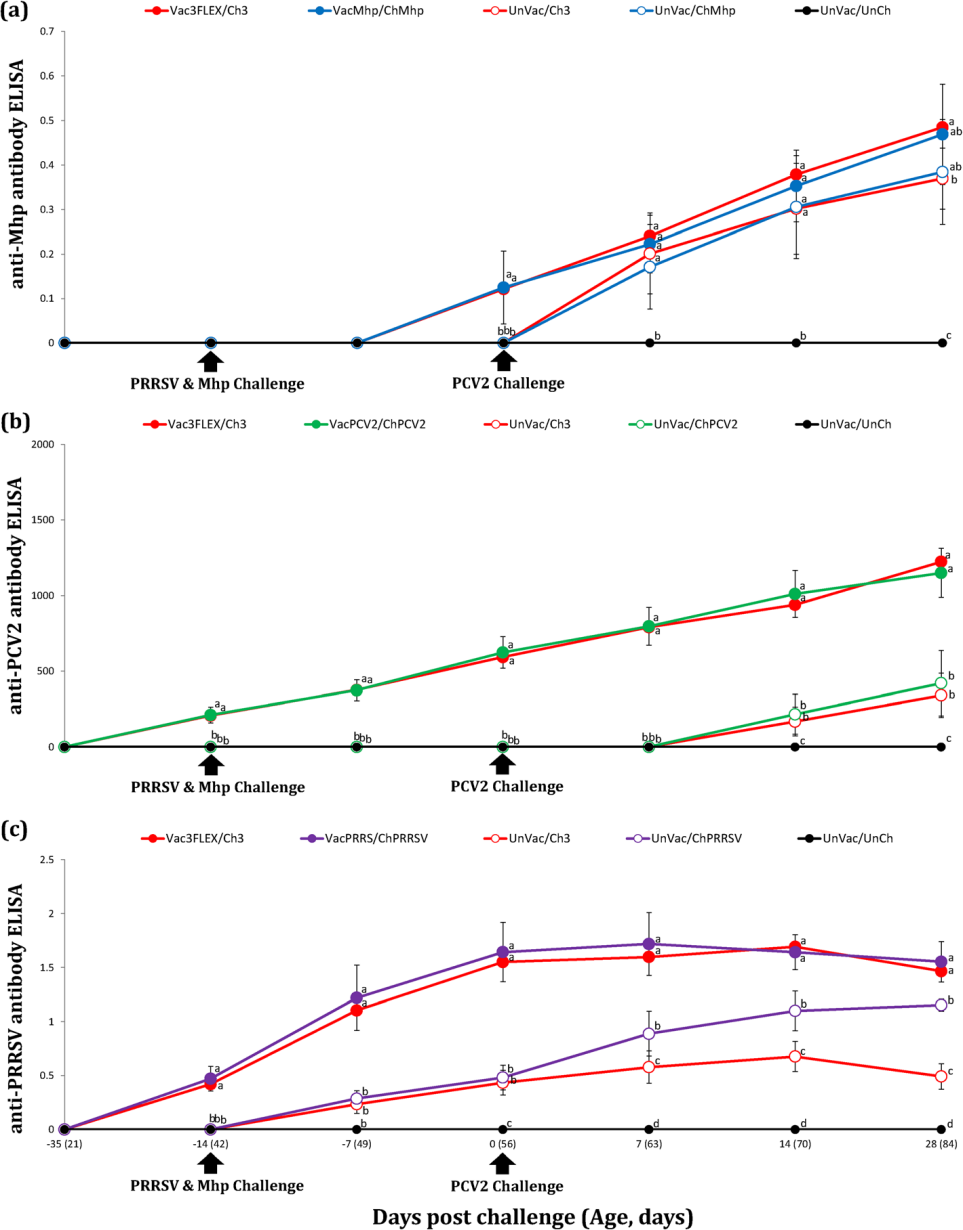
**a** Mean values of the *M. hyopnuemoniae* ELISA antibodies. **b** Mean values of the PCV2 ELISA antibodies. **c** Mean values of the PRRSV ELISA antibodies. Variation is expressed as the standard deviation. Different letters within a sampling point mean statistically significant differences (*P* < 0.05)

Antibody response against *M. hyopneumoniae* was assessed with ELISA. Pigs in the Vac3FLEX/Ch3 and VacMhp/ChMhp groups had a significantly higher (*P* < 0.05) *M. hyopneumoniae* ELISA S/P ratio compared to the UnVac/Ch3 and UnVac/ChMhp groups at 0 dpc. Pigs in the Vac3FLEX/Ch3 group also had a significantly higher (*P* < 0.05) *M. hyopneumoniae* ELISA S/P ratio compared to the UnVac/Ch3 group at 28 dpc (Fig. 3a).

Pigs from the Vac3FLEX/Ch3 and VacPCV2/ChPCV2 groups had a significantly higher (*P* < 0.05) PCV2 ELISA titers compared to the UnVac/Ch3 and UnVac/ChPCV2 groups between − 14 to 28 dpc (Fig. 3b).

The Vac3FLEX/Ch3 and VacPRRS/ChPRRSV groups had a significantly higher (*P* < 0.05) PRRSV ELISA S/P ratio compared to the UnVac/Ch3 and UnVac/ChPRRSV groups from − 14 to 28 dpc. The UnVac/ChPRRSV group had a significantly higher (*P* < 0.05) PRRSV ELISA S/P ratio compared to the UnVac/Ch3 group at 7, 14, and 28 dpc (Fig. 3c). No antibodies against *M. hyopneumoniae*, PCV2, and PRRSV were detected in any of the pigs from UnVac/UnCh group (Fig. 3c). All pigs from all 9 groups were negative for influenza A virus antibodies.

### Interferon-γ secreting cells

**Fig. 4:**
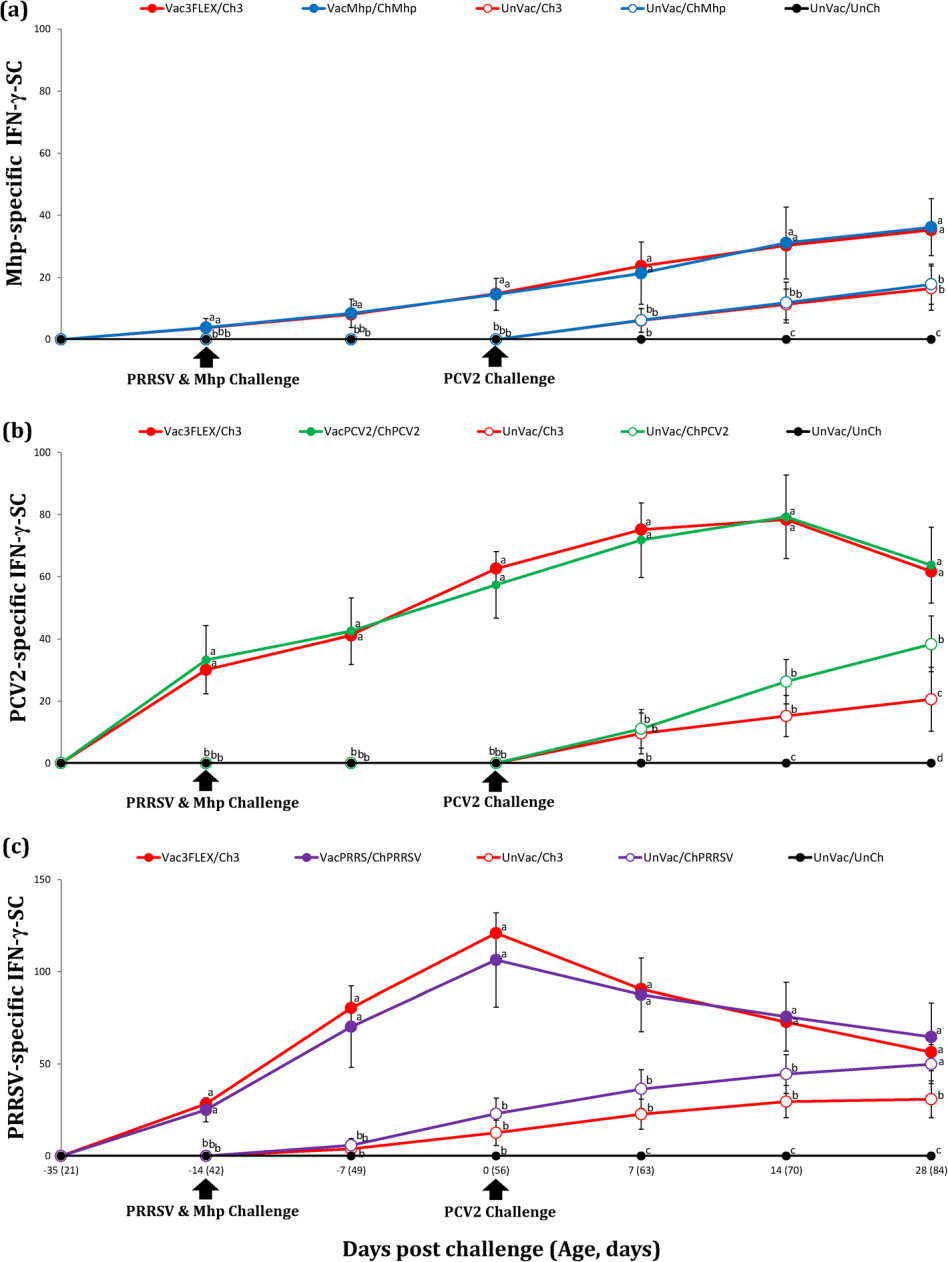
**a** Frequency of *M. hyopneumoniae*-specific IFN-γ-SC/10^6^ PBMC. **b** Frequency of PCV2-specific IFN-γ-SC/10^6^ PBMC. **c** Frequency of PRRSV-specific IFN-γ-SC/10^6^ PBMC. Variation is expressed as the standard deviation. Different letters within a sampling point mean statistically significant differences (*P* < 0.05)

For T cell response the number of *M. hyopneumoniae*-specific IFN-γ-SC was quantified in the PBMC of individual pigs. Pigs from the Vac3FLEX/Ch3 and VacMhp/ChMhp groups had a significantly higher (*P* < 0.05) number of *M. hyopneumoniae*-specific IFN-γ-SC in their PBMC compared to the UnVac/Ch3 and UnVac/ChMhp groups between − 14 to 28 dpc (Fig. 4a).

T cell response was evaluated by comparing the number of PCV2-specific IFN-γ-SC. Pigs from the Vac3FLEX/Ch3 and VacPCV2/ChPCV2 groups had a significantly higher (*P* < 0.05) numbers of PCV2-specific IFN-γ-SC in their PBMC compared to the UnVac/Ch3 and UnVac/ChPCV2 groups from − 14 to 28 dpc. Pigs from the UnVac/ChPCV2 group had a significantly higher (*P* < 0.05) numbers of PCV2-specific IFN-γ-SC in their PBMC compared to the UnVac/Ch3 at 28 dpc (Fig. 4b).

The Vac3FLEX/Ch3 and VacPRRS/ChPRRSV groups had a significantly higher (*P* < 0.05) numbers of PRRSV-specific IFN-γ-SC in their PBMC compared to the UnVac/Ch3 and UnVac/ChPRRSV groups from − 14 to 14 dpc. The Vac3FLEX/Ch3, VacPRRS/ChPRRSV, and UnVac/ChPRRSV groups had a significantly higher (*P* < 0.05) number of PRRSV-specific IFN-γ-SC in their PBMC compared to the UnVac/Ch3 group at 28 dpc (Fig. 4c). The mean numbers of *M. hyopneumoniae*-, PCV2- and PRRSV-specific IFN-γ-SC in the UnVac/UnCh group remained at basal levels (< 20 cells/10^6^ PBMC) throughout the study.

### Pathology

Pigs in the Vac3FLEX/Ch3 and VacMhp/ChMhp groups had significantly lower (*P* < 0.05) macroscopic lung lesion scores, microscopic mycoplasmal and interstitial lung lesion scores compared to the UnVac/Ch3 group at 28 dpc. Pigs from the VacMhp/ChMhp group had significantly lower (*P* < 0.05) macroscopic lung lesion scores and microscopic mycoplasmal lung lesion scores compared to the UnVac/ChMhp group at 28 dpc (Table 2).

**Table 2:** Lung lesion scores in trivalent vaccine mixture and respective monovalent vaccines

Groups	Macroscopic	Microscopic	
	Lung lesion score	Mycoplasmal lesion score	Interstitial lesion score
Vac3FLEX/Ch3	9 ± 4.47 ^bc^	0.6 ± 0.68 ^b^	0.9 ± 0.64 ^b^
VacMhp/ChMhp	5.75 ± 4.94 ^cd^	0.4 ± 0.50 ^bc^	0.1 ± 0.31 ^c^
UnVac/Ch3	42 ± 11.11 ^a^	3.5 ± 0.71 ^a^	3.3 ± 0.82 ^a^
UnVac/ChMhp	12.5 ± 6.35 ^b^	3.1 ± 0.74 ^a^	0.2 ± 0.42 ^c^
UnVac/UnCh	0.5 ± 1.58 ^d^	0 ^c^	0.1 ± 0.32 ^c^
Vac3FLEX/Ch3	9 ± 4.47 ^b^	0.6 ± 0.68 ^b^	0.9 ± 0.64 ^b^
VacPCV2/ChPCV2	1.75 ± 2.45 ^c^	0.05 ± 0.22 ^c^	0.1 ± 0.31 ^c^
UnVac/Ch3	42 ± 11.11 ^a^	3.5 ± 0.71 ^a^	3.3 ± 0.82 ^a^
UnVac/ChPCV2	4.5 ± 3.69 ^bc^	0.2 ± 0.42 ^bc^	0.9 ± 0.57 ^b^
UnVac/UnCh	0.5 ± 1.58 ^c^	0 ^c^	0.1 ± 0.32 ^c^
Vac3FLEX/Ch3	9 ± 4.47 ^bc^	0.6 ± 0.68 ^b^	0.9 ± 0.64 ^c^
VacPRRS/ChPRRSV	4.7 ± 3.36 ^cd^	0.1 ± 0.31 ^c^	0.25 ± 0.44 ^d^
UnVac/Ch3	42 ± 11.11 ^a^	3.5 ± 0.71 ^a^	3.3 ± 0.82 ^a^
UnVac/ChPRRSV	11.5 ± 6.69 ^b^	0.1 ± 0.32 ^bc^	1.5 ± 0.53 ^b^
UnVac/UnCh	0.5 ± 1.58 ^d^	0 ^c^	0.1 ± 0.32 ^d^

Different letters mean statistically significant differences within 5 groups (*P* < 0.05)

Pigs from the Vac3FLEX/Ch3, VacPCV2/ChPCV2, and UnVac/ChPCV2 groups had significantly lower (*P* < 0.05) macroscopic lung lesion scores, microscopic mycoplasmal and interstitial lung lesion scores compared to the UnVac/Ch3 group at 28 dpc. Pigs from the VacPCV2/ChPCV2 group had significantly lower (*P* < 0.05) microscopic interstitial lung lesion scores compared to the UnVac/ChPCV2 group at 28 dpc (Table 2).

The Vac3FLEX/Ch3, VacPRRS/ChPRRSV, and UnVac/ChPRRSV groups had significantly lower (*P* < 0.05) macroscopic lung lesion scores, microscopic mycoplasmal and interstitial lung lesion scores compared to the UnVac/Ch3 group at 28 dpc. The VacPRRS/ChPRRSV group had significantly lower (*P* < 0.05) macroscopic lung lesion scores and microscopic interstitial lung lesion scores compared to the UnVac/ChPRRSV group at 28 dpc. There were no macroscopic and microscopic lung lesions observed in pigs from the UnVac/UnCh group (Table 2).

## Discussion

We have assessed the efficacy of a trivalent vaccine mixture against *M. hyopneumoniae*, PCV2, and PRRSV and compared it to the respective monovalent vaccinated and unvaccinated positive control groups. The trivalent vaccine mixture was able to reduce clinical signs, lung lesions, PRRSV and PCV2 viremia and improve weight gain compared to the unvaccinated positive control groups. The trivalent vaccine mixture, however, did not result in equal protection when compared against each respective monovalent vaccine, with the largest vaccine occurring within PRRSV. Although the PRRS vaccine in this study is widely used, an efficacious, commercially available PRRSV vaccine continues to be a global challenge, as PRRSV variant strains are continuously emerging from highly pathogenic outbreaks (particularly in Asia).

Pigs vaccinated with the trivalent vaccine following a triple challenge significantly improved their growth performance when compared to the unvaccinated control pigs. In contrast, growth performance was better but not significantly different between *M. hyopneumoniae*-vaccinated and control pigs following *M. hyopneumoniae* challenge. These results are consistent with previous findings, where no significant difference on growth performance was observed between *M. hyopneumoniae*-vaccinated and unvaccinated pigs following an *M. hyopneumoniae* challenge [3, 4]. No significant difference on growth performance was also observed between PRRS-vaccinated and control pigs [5]. The possible reason for the lack of statistical significance could be that the growth performance of the pigs vaccinated with the monovalent vaccine was only evaluated for 6 weeks after a single challenge. In addition, growth retardation by a single challenge is likely not very severe. Therefore, the improvement on growth performance by the monovalent vaccines is not as drastic. A triple challenge is typically more severe than a single challenge. In addition, in a field study, infection with *M. hyopneumoniae*, PRRSV, and PCV2 increased the chance for opportunistic secondary bacterial infections which could result in further growth retardation. However, we did not observe a decrease in growth performance due to secondary bacterial infection in this experimental study. Taken together the results suggest that growth performance is an important parameter in evaluating the trivalent vaccine mixture but not as important for evaluating the efficacy of the monovalent vaccines under experimental conditions.

PRDC cannot be controlled without controlling *M. hyopneumoniae* because *M. hyopneumoniae* infections exacerbate lung lesions caused by PRRSV and PCV2 in infected pigs [6, 7]. In addition, previous work has shown that an *M. hyopneumoniae* vaccine can reduce interstitial pneumonia caused by PRRSV [8]. Therefore, control of *M. hyopneumoniae* is the first step to control PRDC caused by the three challenge pathogens used in this study. The trivalent vaccine mixture and monovalent *M. hyopneumoniae* vaccine were able to elicit similar numbers of *M. hyopneumoniae*-specific IFN-γ-SC in vaccinated pigs. This is important since, the cell-mediated immunity as measured by IFN-γ-SC has been shown to play an important role in controlling *M. hyopneumoniae* infection [9, 10]. Induction of cell-mediated immunity is also associated with a significant reduction in the amount of *M. hyopneumoniae* nasal shedding [11]. Vaccination with the trivalent product resulted in a comparable reduction of *M. hyopneumoniae* nasal shedding and lung lesions to that of the respective *M. hyopneumoniae* monovalent.

When comparing the efficacy between the trivalent vaccine mixture and the monovalent PCV2 vaccine, we looked at the cell-mediated immunity elicited by the PCV2 vaccines because it is an important immunity mechanism which contributes to the PCV2 clearance in the blood [12, 13]. In addition, a positive correlation has been reported between PCV2 viremia and the severity of observed lesions [12, 14]. Therefore, induction of IFN-γ-SC and reduction of PCV2 viremia are the critical parameters in evaluating a PCV2 vaccine. In our study, there was no significant difference in the number of PCV2-specific IFN-γ-SC and reduction of PCV2 viremia between the trivalent vaccine mixture and monovalent PCV2 vaccine.

Lastly, we compared the efficacy of trivalent vaccine mixture against PRRSV with that of the monovalent PRRS vaccine and unvaccinated positive control. Reduction in viremia and lung lesions, and induction of cell-mediated immunity, specifically PRRSV-specific IFN-γ-SC which are used for the assessment of antigen-specific T-cell responses in swine [15, 16] are important criteria for PRRSV vaccine evaluation. Despite the fact that the protective role of IFN-γ-SC is controversial [17], correlation between activation of T cell responses and clearance of PRRSV in blood has been previously reported [18, 19]. These data suggest that T cell responses elicited by PRRSV MLV vaccine play a role in the reduction of PRRSV viremia in vaccinated-challenged pigs. In our study vaccination with either trivalent vaccine mixture or monovalent PRRS vaccine, both resulted in the induction of T cell responses and a reduction in the viral load in the blood simultaneously. In addition, there was no statistical difference in the number of PRRSV-specific IFN-γ-SC, levels of PRRSV viremia, and lung lesion scores between trivalent vaccination and monovalent vaccination in pigs.

In this study, we have presented evidence that a trivalent vaccine mixture is efficacious against challenge with three pathogens (*M. hyopneumoniae*, PCV2, and PRRSV) and it is similar to the efficacies of each individual monovalent vaccine against their respective single challenge. Additional interference studies would need to be conducted to establish this ratio and to determine compatibility of the trivalent vaccine mixture compared to the respective monovalent vaccines, especially in regard to the effect on the viability of PRRSV MLV vaccine. In addition, it is difficult to draw a full conclusion from the direct comparison with the respective monovalent vaccines, because of the different challenge inocula between the trivalent vaccine mixture group and the monovalent vaccine groups. Additional test groups of pigs vaccinated with monovalent vaccine followed by a triple challenge or vaccinated with the trivalent vaccine mixture followed by a single challenge with either *M. hyopneumoniae*, PCV2 or PRRSV should be included for further evaluation.

## Conclusions

The results in this study demonstrate that the trivalent vaccine is significantly efficacious against a triple challenge of *M. hyopneumoniae*, PCV2, and PRRSV. The trivalent vaccine mixture, however, did not result in equal protection when compared against each respective monovalent vaccine, with the largest variance occurring within PRRSV.

## Methods

### Pathogens

Three challenge strains were used in this study. These included PRRSV strain SNUVR090851 (PRRSV-2, lineage 1, GenBank JN315685), *M. hyopneumoniae* strain SNU98703, and PCV2b strain SNUVR000463 (GenBank KF871068). Several key reasons played an important role in defining strain selection. The *M. hyopneumoniae* (strain SNU98703) produced lesions typical of *M. hyopneumoniae* found within the peribronchial and peribronchiolar lymphoid tissue hyperplasia in the lungs of infected pigs [20], while the PRRSV (strain SNUVR090851) caused interstitial pneumonia in the lungs of infected pigs [8], and the PCV2b (strain SNUVR000463) caused mild lymphoid depletion in the lymph nodes of infected pigs [21]. A triple challenge using *M. hyopneumoniae* (strain SNU98703), PRRSV (strain SNUVR090851), and PCV2b (strain SNUVR000463) also produced similar clinical symptoms [22].

### Animals

The piglets used in this study were selected based on breeding herd negative serology screening for both PRRSV and *M. hyopneumoniae* prior to purchase. Long term clinical and slaughter history was also taken into account. Piglets selected for the study were 18-days-old at the time of purchase, colostrum-fed, and were Large White, Landrace, and Duroc crossbreds. The piglets were deemed to be clinically healthy upon arrival at 21 days of age. Piglets were screened and found seronegative for the following: PRRSV (IDEXX PRRS X3 Ab test, IDEXX Laboratories Inc., Westbrook, Maine, USA), *M. hyopneumoniae* (*M. hyo*. Ab test, IDEXX Laboratories Inc.), PCV2 (PCV2 Ab Mono Blocking, Synbiotics, Lyon, France), and influenza A virus (Influenza A Ab test, IDEXX Laboratories Inc.). Serum samples were collected and tested for PCV2 and PRRSV by real-time polymerase chain reaction (PCR) [23]. Nasal swabs for *M. hyopneumoniae* were also collected and tested by real-time PCR [23]. All serum and nasal swabs produced negative results.

### Experimental design

The random number generator function (Excel, Microsoft Corporation, Redmond, Washington, USA) was used to randomly divide 130 piglets into 9 groups (Table 3). A minimum sample size per each group was calculated as suggested by Cohen [24] using pwr package in R v.3.5.1 (R Core Team: a language and environment for statistical computing. R Foundation for Statistical Computing, Vienna, Austria, http://www.r-project.org). A 0.05 significance level, 0.4 effect size, and 70% power were used to calculate the minimum number of piglets needed per group. This value was determined as 9.47, therefore, at least 10 piglets were designated per group.

**Table 3:** Experimental design with vaccination and challenge strategies for PRRSV, *M. hyopneumoniae* (Mhp), and PCV2 at different days post-challenge (dpc)

Groups	Vaccination (dpc)	Challenge (dpc)		Necropsy (dpc)
	−35	−14	0	28
Age (days)	21	42	56	84
Vac3FLEX/Ch3	3FLEX	PRRSV/Mhp	PCV2	20 pigs
VacMhp/ChMhp	MycoFLEX	Mhp	None	20 pigs
VacPCV2/ChPCV2	CircoFLEX	None	PCV2	20 pigs
VacPRRS/ChPRRSV	Ingelvac PRRS MLV	PRRSV	None	20 pigs
UnVac/Ch3	None	PRRSV/Mhp	PCV2	10 pigs
UnVac/ChMhp	None	Mhp	None	10 pigs
UnVac/ChPCV2	None	None	PCV2	10 pigs
UnVac/ChPRRSV	None	PRRSV	None	10 pigs
UnVac/UnCh	None	None	None	10 pigs

Blinded personnel (defined as personnel who could not identify the vaccination status of the pigs) performed all actions including the administration of vaccines, administration of the PBS control, recorded observation and pulled defined measurements. The study was conducted at the Seoul National University, Department of Veterinary Pathology which contained a HEPA-filtered isolator. One room contained pigs from each group. The rooms each contained 10 pens with an individual pig housed per pen. Vaccinated groups were randomly assigned to each to include 20 pigs, 10 of which were female and 10 of which were male. These were further split into two rooms (10 pigs per room). Unvaccinated groups were assigned 10 pigs (5 male and 5 female) which were housed in a single room. The random number generator (Excel, Microsoft Corporation) was used to assign pigs to groups and rooms. Several key housing elements were taken into consideration such as both slatted and solid surface pen flooring, and rooms were lit for 12 h/day with the light intensity set to 40 lx in order to simulate daytime. The temperature in each room was also kept at a constant 22 °C. Water was available for piglets to drink freely throughout the day via a nipple drinker which was placed in each pen. Each pen was additionally equipped with a self-feeder which provided access to a standard-balanced, age-appropriate, pelleted feed diet. Playtime stimulation was offered by placing a rubber ball in each pen.

At − 35 days post challenge (dpc, 21 days old), pigs in the Vac3FLEX/Ch3 group were administered one dose (2 mL) of 3FLEX (Boehringer Ingelheim Vetmedica) on the left side of the neck. The 3FLEX vaccine was prepared as follows: MycoFLEX (Serial no. 2730534A) and CircoFLEX (Serial no. 3091124A) were mixed together, and the mixture was then used to rehydrate Ingelvac PRRS MLV (Serial no. 2451180A). This was done in place of the Ingelvac PRRS MLV accompanying vaccine diluent and against the manufacturer’s mixing directions. Pigs in the VacMhp/ChMhp, VacPCV2/ChPCV2, and VacPRRS/ChPRRSV groups were administered one dose of MycoFLEX (1 mL), CircoFLEX (1 mL) and Ingelvac PRRS MLV (2 mL) respectively, on the left side of the neck. The pigs in the UnVac/Ch3, UnVac/ChMhp, UnVac/ChPCV2, UnVac/ChPRRSV, and UnVac/UnCh groups were administered one dose (1 mL) of phosphate buffered saline (PBS, 0.01 M, pH 7.4) as a control.

At − 14 dpc (42 days old), the pigs in the Vac3FLEX/Ch3 and UnVac/Ch3 groups were challenged with two pathogens: PRRSV and *M. hyopneumoniae*. Three milliliters of the PRRSV inoculation containing 1.2 × 10^5^ 50% tissue culture infective dose (TCID_50_)/mL was intranasal administered (1.5 mL per nostril). Five hours later pigs were anesthetized with a mixture of 2.2 mg/kg xylazine hydrochloride (Rompun, Bayer Korea, Seoul, Korea) and 2.2 mg/kg tiletamine hydrochloride and 2.2 mg/kg zolazepam hydrochloride (Zoletil 50, Virbac Korea, Seoul, Korea) by intramuscular injection to prepare for the *M. hyopneumoniae* portion of the challenge. Pigs were then inoculated intratracheally with 7 mL (3.5 mL per nostril) of *M. hyopneumoniae* culture medium containing 10^7^ color changing units (CCU)/mL as previously described [25, 26]. This separation of challenges was done to avoid mixing of the two pathogens which could potentially affect their infectivity. Pigs in the VacPRRS/ChPRRSV and UnVac/ChPRRSV groups were inoculated with the same lot of PRRSV and in the same way as described above, and pigs in the VacMhp/ChMhp and UnVac/ChMhp groups were inoculated with the same *M. hyopneumoniae* lot and in the same manner as described above.

At 0 dpc (56 days old), pigs in the Vac3FLEX/Ch3, VacPCV2/ChPCV2, UnVac/Ch3, and UnVac/ChPCV2 groups were intranasally administered a 3 mL inoculum containing 1.2 × 10^5^ TCID_50_/mL of PCV2. Nasal swabs and blood samples were collected at − 35, − 14, − 7, 0, 7, 14, and 28 dpc from all piglets. At 28 dpc (84 days old), piglets were intravenously sedated with a 1 mL/10 kg dose of sodium pentobarbital and were then euthanized by electrocution using a current of 110 V at a minimum frequency of 60 Hz for a minimum of 3 s [27]. Euthanized piglets were necropsied and tissues were collected and fixed for 24 h in 10% neutral buffered formalin, and embedded in paraffin.

### Clinical observations

Pigs were monitored weekly post-challenge by blinded personnel for changes in physical conditions and clinical respiratory disease symptoms. Respiratory disease severity was scored on a scale ranging from 0 (normal) to 6 (severe dyspnea and abdominal breathing) as previously described [28].

### Average daily weight gain

Pigs were measured for live weight at − 35 dpc (21 days old), 0 dpc (56 days old), and 28 dpc (84 days old). The average daily weight gain (ADWG; grams/pig/day) was analyzed over two time periods: (i) between − 35 and 0 and (ii) between 0 and 28. ADWG was calculated as the difference between the starting and final weight divided by the duration of the stage. Data for dead or removed pigs were also included in the calculation.

### Quantification of PRRSV RNA in blood

RNA was extracted from serum samples to assess PRRSV viremia, as previously described [23]. PRRSV genomic cDNA copies were quantified with real-time PCR for both the challenge and vaccine PRRSV strains [23].

### Quantification of *M. hyopneumoniae* DNA in nasal swabs

*M. hyopneumoniaee* genomic DNA copies were quantified by real-time PCR after DNA was extracted from nasal swabs using a commercial kit (QIAamp DNA Mini Kit, QIAGEN, Valencia, California, USA) [29].

### Quantification of PCV2 DNA in blood

DNA extraction using a commercial kit (QIAamp DNA Mini Kit, QIAGEN) was performed followed by real-time PCR to quantify the PCV2 genomic DNA copy numbers from serum samples [29].

### Serology

Serum samples were tested with ELISA kits for antibodies against the following: PRRSV (IDEXX PRRS X3 Ab test, IDEXX Laboratories Inc.), *M. hyopneumoniae* (*M. hyo*. Ab test, IDEXX Laboratories Inc.), PCV2 (PCV2 Ab Mono Blocking, Synbiotics), and influenza A virus (Influenza A Ab test, IDEXX Laboratories Inc.). Serum samples were considered positive for PRRSV and *M. hyopneumoniae* antibodies if the sample-to-positive (S/P) ratio was **≥**0.4, while serum samples were considered positive for influenza A virus if the sample-to-negative (S/N) ratio was < 0.6, and serum samples were considered positive for PCV2 IgG antibody if the reciprocal ELISA titer was greater than 350 according to the manufacturer’s instructions.

### Interferon-γ secreting cells

The numbers of PRRSV-, PCV2- and *M. hyopneumoniae*-specific interferon-γ secreting cells (IFN-γ-SC) were quantified in peripheral blood mononuclear cells (PBMC) as described by use of the PRRSV, PCV2 and *M. hyopneumoniae* challenge strains respectively [3, 6, 15, 30].

### Pathology

Morphometric analysis of the macroscopic pulmonary lesion has been previously described [28]. Lungs were scored on a total scale of 100 points as follows: 10 points each to the right cranial lobe, right middle lobe, left cranial lobe, and left middle lobe, 27.5 points each to the right caudal lobe and left caudal lobe, and 5 points to the accessory lobe [28].

Microscopic pulmonary lesions were scored for interstitial pneumonia ranging from 0 (normal) to 6 (severe diffuse) [28]. Mycoplasmal pneumonia lesions were scored (0 to 6) based on the severity of peribronchiolar and perivascular lymphoid tissue hyperplasia [6]. All lung section scoring was performed by a blinded (defined by source of the sections) pathologist.

### Statistical analysis

The experimental unit for analysis consisted of data collected from each individual piglet. One-way analysis of variance (ANOVA) and Kruskal-Wallis test were used in this study. ANOVA is a parametric statistical test to analyze the difference between group means, while the Kruskal Wallis test is a non-parametric analogue of ANOVA. ANOVA was used with variables that showed the following: normal distribution such as ADWG, PRRSV RNA, *M. hyopneumoniae* DNA, PCV2 DNA, PRRSV antibody titer, *M. hyopneumoniae* antibody titer, PCV2 antibody titer, and number of IFN-γ-SC. The Kruskal-Wallis test was performed for variables without a normal distribution such as clinical signs, macroscopic lung lesion scores, and microscopic lung lesion scores. When a significant difference existed between the groups, post hoc multiple comparison tests with Tukey’s adjustment was conducted (t-test for ANOVA and Man-Whitney test for Krustal-Wallis analysis) to determine the significant differences between the pairwise groups. A value of *P* < 0.05 was considered significant.

## Data Availability

The datasets used and/or analysed during the current study are available from the corresponding author on reasonable request.

## Abbreviations

ADWG: Average daily weight gain
IFN-γ-SC: Interferon-γ secreting cells
Mhp: *Mycoplasma hyopneumoniae*
PBMC: Peripheral blood mononuclear cells
PCV2: Porcine circovirus type 2
PRDC: Porcine respiratory disease complex
PRRSV: Porcine reproductive and respiratory syndrome virus
